# Cooking, Shopping, and Eating Behaviors of African American and Hispanic Families: Implications for a Culturally Appropriate Meal Kit Intervention

**DOI:** 10.3390/ijerph18189827

**Published:** 2021-09-17

**Authors:** Lauren H. Sweeney, Kaley Carman, Elder G. Varela, Lisa A. House, Karla P. Shelnutt

**Affiliations:** 1 Food Science and Human Nutrition Department, University of Florida, Gainesville, FL 32611, USA; lheadrick@ufl.edu; 2 Department of Family, Youth and Community Sciences, University of Florida, Gainesville, FL 32611, USA; kmialki@ufl.edu; 3 Department of Health Education and Behavior, University of Florida, Gainesville, FL 32611, USA; elder89@ufl.edu; 4 Food and Resource Economics Department, University of Florida, Gainesville, FL 32611, USA; lahouse@ufl.edu

**Keywords:** meal kit, low-income, cooking, family

## Abstract

Families with low incomes face barriers to preparing healthy meals, including decreased food access and limited time, and may turn to fast, low-quality, and inexpensive foods. Affordable and accessible meal kits may reduce these barriers. The objective of this study was to explore the cooking, eating, and shopping behaviors of African American (AA) and Hispanic participants living in the United States with low incomes and determine the knowledge of and preferences for a culturally appropriate meal kit intervention. Trained researchers conducted focus groups using a semi-structured questionnaire with AA and Hispanic food preparers with low incomes. Participant cooking, eating, and shopping behaviors and knowledge of and preferences for a culturally appropriate meal kit intervention were evaluated using thematic analysis. AA participants (n = 16) reported cooking on average 2 to 3 days per week and more often on weekends. Hispanic participants (n = 15) reported cooking 5 days per week and more often during the week. Both groups identified cost as the number one consideration when shopping. Most were unfamiliar with meal kits but indicated they would try an affordable meal kit. AA and Hispanic participants differed in their cooking, eating, and shopping behaviors but were equally interested in trying meal kits if affordable and culturally appropriate.

## 1. Introduction

Individuals who live in poverty, or those with incomes at or less than 185% of the federal poverty line, face multiple barriers to healthy eating, including food insecurity [1]. According to the most recent United States Department of Agriculture (USDA) data, an estimated 10.5% of U.S. households face food insecurity [1]. The USDA defines food insecurity as a reduction in the quality, variety, or desirability of a diet or a disrupted eating pattern and reduced food intake [1]. Those with the highest prevalence of food insecurity include African American (AA) and Hispanic individuals [2]. Food insecurity negatively affects diet quality, leading to health problems, including overweight and obesity, diabetes, and heart disease [3,4]. A systematic review assessing the relationship between food security and dietary quality found that adults with food insecurity consume fewer fruits, vegetables, and dairy foods compared to adults without food insecurity [5].

As Americans consume 68% of their calories at home and spend 57% of their food budget for food at home, this may be the most appropriate location for a nutrition intervention [6,7]. To create a home-based dietary intervention that is affordable for populations with lower income, it is important to understand the food environment of these families, including opportunities and barriers for healthy eating at home. A study with 1500 families with low to middle income that solicited information on shopping, cooking, and mealtime habits found that participants went grocery shopping once per week and ate dinner at home 4 days per week [8]. Notably, participants identified food cost, time, and conflicting schedules as barriers to cooking meals at home but also commented that they feel they can overcome these hurdles to cook healthy meals for their families [8]. A cross-sectional study of 100 individuals with low income in Texas identified the following barriers to healthy eating: cost, time, transportation, lack of knowledge of healthy foods, lack of cooking skills, and distaste for healthy foods [9]. These data suggest that there is a need for an affordable, simple, and quick intervention to remove the stated barriers to cooking healthy meals at home.

Meal kits may be a way to reduce these barriers [10]. A meal kit is a box or bag of perishable and non-perishable ingredients for one or more meals based on a recipe that requires minimal preparation that also includes a step-by-step recipe card describing how to cook each meal at home [11,12]. The $10.26 billion meal kit industry continues to gain popularity due to the convenience it provides by eliminating the process of meal planning and grocery shopping [12,13]. Consumers of home-delivered meal kits tend to be between the ages of 25 to 44 years old, partnered or married with children in the house, have an advanced degree, a full-time job, and have a household income of at least $100,000 [12]. While individuals with low income cannot use SNAP benefits to purchase traditional home-delivered meal kits, grocery stores are also starting to offer these one-stop-shop meal kits, which are a SNAP-eligible purchase [12].

Few studies have been conducted on the impact of meal kits. Oberle et al. found that meal kits may be a way to reduce barriers to cooking at home [10]. Families with adolescents who have obesity reported positive impacts on barriers to cooking and eating healthy when provided meal kits as part of a pediatric weight management program [10]. Additionally, studies conducted with meal kit recipients in New Zealand found meal kits to positively impact diet behaviors, general well-being, and family relationships [11,14]. Current literature is lacking regarding the impact of meal kits on families with low income. The objectives of this study were to explore the cooking, eating, and shopping behaviors of AA and Hispanic participants with low income and to determine the knowledge of and preferences for a culturally appropriate meal kit service. The information from this study will be used to inform the development of a culturally appropriate meal kit intervention for minority families with low income.

## 2. Materials and Methods

### 2.1. Participants

A total of six focus groups were conducted with two separate racial/ethnic groups (three focus groups per racial/ethnic group). Group 1 was a convenience sample of AA participants recruited in-person from a church in a predominately low-income neighborhood in Gainesville, Florida, United States. United States Census tract poverty rate data were used to qualify the neighborhood as low income [15]. Inclusion criteria were (1) AA, (2) 18 years of age or older, and (3) primary food preparer in the household. Group 2 was a convenience sample of Hispanic participants recruited in-person from a community resource center predominately serving participants who are low income in Hollywood, Florida, United States. Inclusion criteria were (1) Hispanic, (2) 18 years of age or older, and (3) primary food preparer in the household. Group 2 focus groups and surveys were conducted in Spanish, the participants’ native language. All focus groups were conducted in groups of five or six at the site in which they were recruited, and participants were divided among groups based on time in which they arrived at the site. The study participants, young children of the participants, and the researchers were the only ones present at the time of data collection. Three focus groups with each racial/ethnic group were sufficient for data saturation, or the point in which no new themes emerged regarding knowledge and preferences for meal kit [16]. Focus groups lasted approximately 45 min and were audio recorded and transcribed verbatim. All participants received $25 for participation. This study was approved by the University of Florida’s Institutional Review Board, and all participants gave informed consent.

### 2.2. Data Collection

A semi-structured questionnaire (Supplementary S1) was designed by the researchers to collect information on food purchasing, preparation, and consumption behaviors, as well as meal kit perceptions. Researchers internally reviewed the questionnaire for clarity. Focus group moderators were trained to collect data uniformly, and focus group sessions were recorded for data analysis purposes. Focus group sessions were conducted in July 2019. Three researchers participated as focus group moderators for Group 1, and two researchers for Group 2. In addition to data collected by focus group moderators, participants also completed a brief demographic survey.

### 2.3. Data Analysis

Descriptive statistics of the demographic data were summarized using IBM SPSS Statistics for Macintosh, version 26 (Armonk, NY, USA). Data from the focus groups with the Hispanic participants were translated from Spanish to English by the researcher who led the focus group sessions and is a native Spanish speaker. Participants’ comments were rearranged and categorized by interview protocol question. Two researchers with experience coding focus group data categorized the data from each question into thematic units and identified similarities and differences among participants for each question [17]. Themes were developed individually and then cross-checked by a second researcher until consensus was reached. Finally, quotes highlighting each of the major themes were identified. While questions were presented to all participants, response rates differ by question as not all participants chose to answer.

## 3. Results

### 3.1. Sociodemographic Characteristics

Focus group demographic data are presented in Table 1. No recruited participants refused to participate or dropped out. Briefly, 29/31 participants were female with an average age of 44 ± 15 years old. Participants from Group 1 (n = 16) identified as non-Hispanic and AA, while Group 2 (n = 15) participants identified as Hispanic. While not formally asked, participants from Group 2 reported eight countries of origin, including Argentina, Columbia, Cuba, Dominican Republic, Mexico, Peru, Uruguay, and Venezuela. Additionally, Group 1 had on average three adults living in the household, while Group 2 had on average four adults living in the household.

### 3.2. Food Purchasing Behaviors

When asked what factors they consider when deciding what foods to purchase, a majority of participants from both groups stated cost as the number one consideration. One Hispanic participant said, “I see the specials of the week. What is the cheapest week specials, that is what I buy”. AA participants (3/8) also reported the cost of healthy food as a major barrier for preparing healthy meals at home. Two of the AA participants reported eating less than they felt they should in the past year because there was not enough money to purchase food, whereas eight of the Hispanic participants reported eating less than they felt they should for the same reason. In addition to cost, AA participants also identified nutrition (4/15), available foods or “what is already in the pantry” (4/15), and what the kids will eat (3/15), as playing a role in what is purchased; whereas Hispanic participants also mentioned quality (3/13) and nutrition (4/13). One AA participant responded that when shopping she considers “the ingredients on the back” because she has “to watch for sodium”.

### 3.3. Food Preparation Behaviors

Weekday and weekend meals differed considerably between AA and Hispanic participants. AA participants (7/11) reported cooking on average two to three days per week and more often cooking on weekends, with one participant stating that “There’s not as much thought put into the, I don’t think, into the weekday meals versus on the weekend when you have more time”. This statement was reinforced by another AA participant who stated that “Weekday meals are faster”. Conversely, Hispanic participants (9/15) reported cooking five days per week and more often during the week with Hispanic participants stating “I try to wait until it is Saturday or Sunday to eat out and if we have something to do because we have more free time” and that “Sundays are for eating out”. Hispanic participants (6/15) who did not cook evening meals at home during the week preferred a larger lunch and either a small dinner or no dinner at all.

Over the past week, AA participants reported eating dinner together three nights per week, whereas Hispanic participants reported eating together as a family six nights per week. Only six of the AA participants reported eating dinner together five or more nights per weeks, as compared to 12 of the Hispanic participants. When asked on the demographic questionnaire if they look forward to eating meals with their family, most AA (12/16) and Hispanic (12/15) participants said they agreed or strongly agreed with that statement (data not shown). The remaining participants from both groups strongly disagreed to looking forward to eating meals with their families.

When asked about barriers to cooking evening meals during the week, both AA (8/10) and Hispanic (7/14) participants reported time as the main barrier to cooking evening meals at home during the week. A majority of AA participants (7/10) reported spending 30 min to one hour preparing and cooking dinner during the week. On the other hand, about half of the Hispanic participants reported spending more than one hour preparing and cooking an evening meal during the week, while the other half reported spending about 30 min. To reduce the time spent cooking during the week, participants from both groups reported using a crock pot to prepare ahead of time and cooking enough to have leftovers for multiple days.

### 3.4. Food Consumption Behaviors

When asked how often they ate at least two different fruits and two different vegetables at home, participants reported an average of four days each week. Only 2/16 AA and 5/15 Hispanic participants reported eating at least two different vegetables at home every day. Additionally, only 3/16 AA and 1/14 Hispanic participants reported eating at least two different fruits at home every day. Food preference, on the other hand, varied greatly between groups, and even between participants within the same focus group. AA participant food preferences varied greatly based on “the way we were raised” with one participant stating that her older fiancé “likes chicken feet, or oxtails, or neck bones, but I don’t like them”. Hispanic participant preferences varied based on country of origin with one participant explaining how “in my country in Colombia, you call it Sudado de Pollo. But my husband is from Uruguay, so they eat a lot of pasta”, and how because of food preference due to where they were raised, she has to make “two meals so: rice and pasta because my mother-in-law does not eat rice because they are not used to rice at all”. About half of the participants in both groups elicit feedback from other family members (e.g., kids, spouse, parent) on what to eat, but 5/10 AA and 9/13 Hispanic participants said they ultimately decide what is for dinner.

### 3.5. Perception of Meal Kits

None of the participants in either group had ever tried a meal kit. While some had heard of meal kits or knew someone who had tried them, most were unfamiliar with the concept. One AA participant seemed skeptical of the concept stating “Basically, the package comes, and you open it and I’m [going to] eat this tonight… it just doesn’t seem real to me”.

After explaining the concept of meal kits and how they work to the participants, all AA and Hispanic participants said they would try a meal kit if it was affordable. Participants found it difficult to estimate how much they would be willing to pay for a meal kit that feeds a family of four. AA participants provided ranges of $10 to $30 per meal and $40 to $120 for three meals, each with four servings. Hispanic participants were more hesitant to provide a range but preferred $20 to $90 for three meals to feed a family of four. Another Hispanic participant was skeptical of the food quality in a box and therefore could not suggest a price stating “Until you do see what the box brings, and what kind of meals you are getting, you cannot put a price on it. Depends on the quality of the chicken or meat or fish”.

To reduce the cost of the traditional meal kit, which is delivered to the door, participants were asked if they would be willing to pick the meal kit up from a central location. A majority of both AA (8/9) and Hispanic participants (10/15) would be willing to pick up the meal kit, with lack of convenience deterring other Hispanic participants. AA participants said they would pick up from a location nearby, such as a church, food bank, or library.

Both groups said the meal kits could help save time and could be good for health. One AA participant who was familiar with the meal kit concept mentioned how it “cuts down on time going to the grocery store” and “it doesn’t take too much preparation time, [so] it saves that time for family time”. Responses varied among AA and Hispanic participants when asked about maximum preparation and cook time; however, all participants cited a range between 20 min to one hour. A majority of AA participants preferred 30–45 min, whereas Hispanic participants preferred an average of 30 min or less of total preparation and cook time.

When asked what would stop them from trying the meal kit, 4/9 AA and 7/14 Hispanic participants said cost. Additional concerns cited were quality, portion sizes, food safety, food preferences/picky eaters, and taste. A Hispanic participant expressed concern that food preferences “depend on how I feel” and that a “box is not beneficial” if she did not “want to cook what the box brings”. One AA participant did not “want it coming through the mail, period” and would rather “go to the farmers market or something like that”, and another AA participant stated that she was “too traditional to even think like that”. A Hispanic participant expressed concern regarding meal kits containing portion sizes that may be too small, stating: “The portions must be small, and you know Hispanic men… they eat a lot. If I’m going to pay a lot of money and the box is not going to bring enough food, it’s not convenient”.

All participants (31/31) agreed that a box containing perishable ingredients would be preferred; however, some AA participants (6/16) also said they wanted the box to include non-perishable ingredients, whereas all Hispanic participants (15/15) only wanted perishable ingredients. All participants also agreed that the boxes would need to contain food the whole family would eat (both adults and children), but that they would be willing to try new foods. One participant from the AA focus groups stated that “I’m willing because of my daughter. When I try something new, she’s like ‘mom, it’s good’ so she loves trying something new”. Additionally, one Hispanic participant stated “If you are going to stop going to the supermarket to buy the boxes, it’s super important [that it feeds everyone] because if it’s not going to be something that the whole family enjoys we’ll have to leave the box and continue as usual”.

## 4. Discussion

The objectives of this study were to explore the cooking, eating, and shopping behaviors of AA and Hispanic participants and to determine the knowledge of and preferences for meal kits. The information from this study was used to inform the development of a meal kit intervention for minority populations with low income. While Hispanic participants were on average 10 years younger than AA participants, both groups had a similar number of people in the household and averaged two children in the household. A majority of participants from both groups identified themselves as the primary grocery shopper and food preparer in their household. While most Hispanic participants had a high school diploma, most AA participants had an associate’s degree or technical school degree. These differences in education may be due to the fact that many of the Hispanic participants were likely first- or second-generation immigrants based on their participation in and utilization of services at the location in which they were recruited.

Both AA and Hispanic participants identified cost as the number one factor when determining what to purchase when shopping. Participants were prompted with a list of possible factors that determined shopping purchases, with cost being listed first, so it is possible this contributed to all participants selecting this factor. Of note is that these results support previously published results from a 2012 study in which 48 AA and Hispanic women with children were interviewed about their food purchasing habits and cost was also found to be the number one factor when making food purchasing decisions [18]. Unlike the current study, participants in the 2012 study also reported convenience as a major deciding factor when making purchasing decisions, often choosing non-perishable frozen or canned items in place of fresh fruits and vegetables [8]. Both AA and Hispanic participants in the current study requested perishable foods in the meal kits, with Hispanic participants preferring only perishable foods. While the amount of time spent cooking was not recorded in the 2012 study, Hispanic participants from the current study spent an average of one hour preparing dinners during the week, which may explain why they prefer fresh fruits and vegetables in a meal kit that take longer to prepare, instead of processed produce.

Hispanic families reported eating evening meals together more often with 12/15 stating that they ate dinner together five or more nights per week compared to 6/16 of AA families. Additionally, all participants in this study stated that they cooked dinner at home on weekdays three to five days per week. According to the 2012 It’s Dinnertime Report, 67% of families from all backgrounds ate dinner together five or more nights per week, and 78% of families ate at home five or more days a week [8]. Food plays a central role in Hispanic culture, meaning families often prepare meals from scratch and eat family meals together more often than non-Hispanic whites and blacks [8,19,20]. AA participants identified more barriers to eating evening meals as a family, including time and cost, possibly factors resulting in fewer evening meals together each week. Time and cost are similarly reported as barriers in the 2012 It’s Dinnertime Report [8]. Also noteworthy are the 7/31 participants who strongly disagreed to looking forward to eating meals with their families. While the reason for this is unclear, negative feelings towards family mealtime may reduce the number of days families cook and eat at home.

The variability in food preferences between the AA and Hispanic groups was also noteworthy, especially as it relates to creating culturally appropriate dietary interventions. Differences in meal preferences in AAs tended to be generational, whereas Hispanic meal preferences differed based on country of origin. While there were generational differences among the AA participants, there was also the number of foods that were consistent among AA participant’s food preferences, a consistency that could be incorporated into a culturally appropriate meal kit. Creating a similar meal kit for Hispanic individuals from different countries, however, would prove more challenging as food preferences vary widely by geographic region. Hispanic participants recruited for this study, representing eight different countries, were likely first- or second-generation immigrants and may not have experienced the same level of acculturation as American-born Hispanic families. As the participants’ food preference reflected traditional dishes from their country of origin, nutrition interventions such as meal kits for these families would need to consider culture and food preferences in order to be most accepted. Culturally sensitive interventions have been shown to be more effective than usual care, as shown in Barrera’s culturally adapted diabetes intervention for Latinas [21,22]. While there is a need to be sensitive to cultural preferences when designing a diet intervention, such as a meal kit, it is also worth noting that participants overwhelmingly said they would be willing to try new foods, illustrating the need for further investigation in this area. Meal kits may provide participants with a cost-effective method for trying new foods as meal kits provide small portions of the ingredients, rather than, for example, an entire bottle of spices.

While previous studies have assessed the acceptability of meal kits as a weight management technique and with populations outside of the U.S., to the best of our knowledge, this is the first study to determine the feasibility of utilizing meal kits as a nutrition intervention in AA and Hispanic populations with low income [10,12]. Results from the meal kit questions of the focus groups suggest that meal kits are a new concept for participants, one that may require more consumer education prior to successful implementation with minority audiences with low income. A study on the acceptability of meal kits with adolescents who have obesity and their caregivers found meal kits promoted cooking at home, saved time, and were seen as an acceptable weight-loss intervention [10]. Similarly, our study found that most participants were open to the idea of trying meal kits, with major concerns centered around cost and quality (freshness and food safety). Traditional meal kits that are delivered to the home cost on average from $5–$15 per serving [23]. Due to the high cost per serving and inability to use SNAP benefits on home-delivered meal kits, this is likely not an option for audiences with low income. However, to save money and create a more cost-effective intervention, participants were willing to pick up the meal kits from a central location, such as a church, food pantry, or library. Grocery stores are also starting to offer these one-stop-shop meal kits, which can be purchased using SNAP benefits, another alternative to the traditional meal kits [12]. Picking up from a centralized location, whether it be a church or grocery store, would also ease food safety concerns. These concerns are not unfounded. Data shared at the 2017 Food Safety Summit and from the Centers for Disease Control and Prevention highlight how the lack of proper food safety procedures among some home-delivered meal kits resulted in an increased chance of food borne illness due to improper handling and chilling [24,25]. By picking meal kits up from a centralized location, the foods can be refrigerated until distribution, allowing participants to enjoy the convenience of meal kits with a reduced risk of food safety concerns.

Participants also questioned portion sizes, taste, and how their individual food preferences would be considered. Hispanic participants cited their desire to cook foods not included in the meal kit as a possible barrier to trying the meals kits. As traditional meal kits most often include three meals a week, notifying families that meal kit services still allow for flexibility to make their own meals is an important educational component. Additionally, a number of meal kit services currently on the market allow consumers to choose the meals they receive each week, further ensuring the meals account for individual food preferences [12]. Based on the findings from this study, previous meal kit studies, and current practices from commercial meal kit studies, a list of recommendations for future meal kit interventions aimed at improving food access and diet quality have been created. Briefly, consumer education is needed to inform individuals with low income about the concept of meal kits and about food safety considerations. Meal kits targeting this segment of the population should be affordable, preferably eligible for purchase using SNAP benefits. Recipes included should continue to be quick to prepare and may include educational resources of common cooking techniques. Finally, the recipes included in the meal kits should also be appropriate for minorities and populations with low incomes who are disproportionately at risk for diet related diseases, such as hypertension and diabetes [26].

This study has several strengths and limitations. One strength includes the use of trained moderators conducting the focus groups in the participant’s native language. Additionally, a strength of this study is the innovative nature of the data collected on meal kits, which will provide the foundation for a future meal kit intervention with an audience with low income. Limitations of this study include the inability to pilot test the semi-structured questionnaire with the target population prior to the focus groups and the use of small convenience samples with only two racial/ethnic groups. The sample size was limited due to the funding available to conduct the study. Finally, in addition to federal food assistance usage, future studies should ask for income data to qualify participants as low income. Participants were all recruited from facilities that support individuals with low income or those that reside in low-income neighborhoods. While it was expected that a high percentage of participants were receiving federal food assistance and therefore considered low income, only 42% of study participants actually reported receiving federal food assistance. It is possible that participants underreported their enrollment in federal food assistance programs as documented by the Urban Institute [27].

## 5. Conclusions

Results from this study show that both AA and Hispanic families prioritize cooking and eating together as a family but may encounter barriers such as cost and time. Meal kits are an innovative way to address these barriers but were a new concept for these participants. Both groups may benefit from utilizing meal kits as a way to quickly and conveniently eat more healthful dinners at home. Both groups identified a lack of knowledge regarding meal kits but a willingness to try them if affordable. While there are concerns related to cost, quality, and food preferences, these could be addressed by understanding the audience for which the meal kits are targeting and altering the method in which participants receive the meal kits to reduce cost while maintaining food safety. Due to concerns over taste, portions, and the desire to cook food in addition to what is provided in the meal kit, additional education about the meal kits in both populations is warranted. If implemented as a nutrition education intervention, the meals kits should be tailored to account for taste and cultural preferences. This study was completed before the COVID-19 pandemic. Since COVID-19, people have been cooking more at home, suggesting meal kits may be more favorable than before [28]. Meal kits may be a unique, pragmatic approach to improving diet quality of families with low income if the service is affordable, maintains high nutrition standards while accounting for dietary preferences of the target audience, and is easily accessible.

## Figures and Tables

**Table 1:** Participant sociodemographic characteristics.

Characteristic	Group 1 (n = 16)	Group 2 (n = 15)	Combined (n = 31)
Ethnicity, n (%)			
Non-Hispanic	16 (100)	0 (0)	16 (52)
Hispanic	0 (0)	15 (100)	15 (48)
Race, n (%)			
White	0 (0)	11 (73)	11 (35)
African American	16 (100)	1 (7)	17 (55)
Native Hawaiian or Other Pacific Islander	0 (0)	2 (13)	2 (6)
Unspecified	0 (0)	1 (7)	1 (3)
Sex, n (%)			
Female	16 (100)	13 (87)	29 (94)
Male	0 (0)	1 (7)	1 (3)
Unspecified	0 (0)	1 (7)	1 (3)
Age (years; mean ± SD)	49 ± 18	39 ± 11	44 ± 15
Highest level of education, n (%)			
Less that high school	0	2 (13)	2 (6)
High school graduate	4 (25)	5 (33)	9 (29)
Some college	2 (13)	2 (13)	4 (13)
Associate degree/technical school graduate	7 (44)	3 (20)	10 (32)
Baccalaureate degree	2 (13)	3 (20)	5 (16)
Advanced college degree	1 (6)	0	1 (3)
Number of people in the household (mean ± SD)	3 ± 2	4 ± 1	3 ± 1
Number of people in the household (range)	1–6	2–6	1–6
Receive federal food assistance, n (%)	6 (38)	7 (47)	13 (42)
Primary grocery shopper in the household, n (%)	13 (81)	12 (80)	25 (81)
Prepares at least 50% of meals in the household, n (%)	14 (88)	13 (87)	27 (87)

SD = Standard deviation; percentages may not equal 100 due to rounding.

## Data Availability

The data in this study are available upon request by the corresponding author.

## Supplementary Materials

The following are available online at https://www.mdpi.com/article/10.3390/ijerph18189827/s1.
