# Supplementary material for: Cooking, Shopping, and Eating Behaviors of African American and Hispanic Families: Implications for a Culturally Appropriate Meal Kit Intervention
Source: Int J Environ Res Public Health. 2021 Sep 17;18(18):9827. doi: 10.3390/ijerph18189827 (PMC8466649; doi:10.3390/ijerph18189827)
Supplement: Supplementary file 1 [file ijerph-18-09827-s001.zip › ijerph-1326396-supplementary.pdf]

International Journal of
Environmental Research and Public Health

MDPI

MODERATOR'S GUIDE

FOCUS GROUP INTERVIEW – FOOD BOX/PREPARERS

DISCUSSION OF EVENING MEALS (Approximately 30 minutes)

1. **Tell us about a typical evening meal during the week.**
2. **Do you eat at home?**

   2a Who prepares?

   2b How often are you cooking at home (days per week)?

   2c How much time do you spend preparing the meal?
3. **Do you eat with others?**

   3a Do you eat together as a family?
4. **Do you prepare your meal or does some else prepare it?**
5. **Do you purchase your meal already prepared? How does it work?**
6. Who decides what you are cooking? You? Your kids? Spouse? Another member of the household?

   6a Do you all eat the same foods?
7. **What are your favorite meals?**
8. **Are weekend meals any different? If so, how?**

Choosing foods:
What are the main things you think about when deciding what to purchase/prepare?

- Cost
- Nutrition
- Time
- Ease of preparation
- What the kids/family likes
- What is on sale this week
- Habits/tradition/skill level

How willing are you/your family to try new foods?

What are some barriers to preparing food at home?

- Cost
- Time
- Knowledge/Ability
- Equipment
- Access to grocery store/transportation
- Storage for fresh food at home

We are exploring different ways to help families prepare food at home. You may have heard of these relatively new companies that are delivering meals in a box, like Blue Apron or Hello Fresh. These companies mail a box of food to a person's home that contains ingredients for several meals. The person typically selects how many people the meals will feed and may have some choice as to what the meals are. The box is delivered to the house weekly, and the ingredients are in pre-portioned amounts, with a recipe card. The person then has to do some minimal preparation, like cutting or chopping vegetables and meat, as well as cooking the meal.

- What do you think about this type of meal service?
- Would you ever try something like this?
- What would keep you from trying? What would be concerns about trying this?
- How important are types of food in the box, or would you be willing to try new things?
- What would the box need to have for you to want to try something like this?
- What is the maximum time you would want the average recipe to take to prepare?
- How important is it that the meal be something everyone in the family would eat (parents and children)?
- In addition to a printed recipe, would you like to see a video of the meal being prepared? If so, would you want a link to a video, an app on a phone, how would you watch the video?
- Would you consider a box that contains fresh fruits and vegetables (that would take some preparation), or would you prefer shelf stable only?
- Would you be willing to try a box like this if it were affordable?
- How much would you pay for a box that provided 3 meals per week for a family of four?
- To reduce cost, would you be willing to pick up a box like this at a central location?
- If so, what types of locations (churches, food bank, schools) would you be willing to go to in order to pick up a box like this.
